# Optimal cut-off values of visceral fat area for predicting metabolic syndrome among patients with ischemic stroke: a cross-sectional study

**DOI:** 10.3389/fneur.2024.1398907

**Published:** 2024-08-02

**Authors:** Xueyan Lu, Jing Wang, Huijie Sun, Dandan Liu, Xiuli Yan, Zhuo Liu

**Affiliations:** ^1^Reproductive Medical Center, Tangdu Hospital, The Fourth Military Medical University, Xi’an, China; ^2^School of Nursing, Xi’an Siyuan University, Xi’an, China; ^3^Cadre Ward, The First Hospital of Jilin University, Changchun, China; ^4^Physical Examination Center, The First Hospital of Jilin University, Changchun, China; ^5^Department of Neurology, The First Hospital of Jilin University, Changchun, China

**Keywords:** visceral fat area, abdominal obesity, metabolic syndrome, ischemic stroke, cut-off value

## Abstract

**Objectives:**

The prevalence of metabolic syndrome (MetS) among patients with ischemic stroke is relatively high. The visceral fat area (VFA) is a predictor of MetS. This study aimed to estimate sex-specific optimal cut-off values of VFA and MetS risk factors among patients with ischemic stroke.

**Methods:**

A cross-sectional study including 851 patients with ischemic stroke was conducted between March 2019 and January 2020 in a tertiary hospital in the northeast of China. VFA was measured using the dual bioelectrical impedance method. Binary logistic regression analysis was used to investigate MetS risk factors, and the VFA cut-off value was assessed using receiver operating characteristic curve analysis.

**Results:**

The overall prevalence of MetS was 43.4%. After adjusting for potential confounders, female sex (odds ratio [OR] = 2.86, *p* < 0.001), the presence of visceral obesity according to VFA (OR = 7.45, *p* < 0.001), being overweight (OR = 2.75, *p* < 0.001) or obesity (OR = 6.00, *p* < 0.001) were associated with an increased risk of MetS. The correlation between VFA and MetS in patients with ischemic stroke was strongest with cut-off values of 104.3 cm^2^ (sensitivity 73.0%, specificity 83.1%) for men, and 94.1 cm^2^ (sensitivity 70.9%, specificity 72.9%) for women.

**Conclusion:**

MetS affected approximately a half of patients with ischemic stroke. Female sex, visceral obesity, and body mass index were independent risk factors for the development of MetS. Sex-specific reference values for VFA are proposed for the prediction of incident MetS in patients with ischemic stroke.

## 1 Introduction

The absolute number of incident and prevalent strokes has increased worldwide over the past 20 years. According to the results of the Global Burden of Disease Study 2019, there were 3.94 million new stroke cases in China in 2019 (1). Obesity has long been suggested as a major controllable risk factor for ischemic stroke. The prevalence of overweight and obesity based on body-mass index (BMI) among Chinese adults exceeds 50%, with the fastest increase in BMI reported between 1990 and 2019 (2, 3). However, it remains unclear whether obesity itself is a risk factor for ischemic stroke. A recent cohort study conducted over 12 years found that the risk of ischemic stroke was associated with the metabolic consequences of obesity (4). The American Association of Clinical Endocrinologists/American College of Endocrinology guidelines also recommended a complications-centric approach be used to assess and treat obesity (5).

Metabolic syndrome (MetS) is the constellation of multiple vascular risk factors. It involves at least three out of five metabolic conditions, including hypertension, glucose intolerance, abdominal obesity, decreased high-density lipoprotein cholesterol (HDL-C), and increased triglyceride (TG) levels, which are also well-established risk factors for ischemic stroke. The prevalence of MetS in patients with ischemic stroke is between 32.5 and 76.8% according to different diagnostic criteria (6, 7). MetS has been shown to be associated with an increased predisposition to stroke, stroke severity, poor prognosis and stroke recurrence (4, 7–10).

The World Health Organization reported that the relative distribution of excess fat could be used to determine risks of cerebrovascular event more effectively than the total amount of fat (11). As the gold standard for the diagnosis of abdominal obesity, the visceral fat area (VFA) accurately reflects visceral fat accumulation (12). Abdominal obesity, characterized by the preferential deposition of fat in the internal visceral region, resulting in an “apple-shaped” figure, has a significant causal association with ischemic stroke (13, 14).

Therefore, VFA may be more suitable for the assessment of abdominal obesity which can accurately identify the risk of MetS in patients with ischemic stroke. This study aimed to estimate sex-specific optimal cut-off values of VFA and MetS risk factors among patients with ischemic stroke in China.

## 2 Methods

### 2.1 Study design and participants

This observational cross-sectional study was conducted in the stroke center of The First Hospital of Jilin University located in northeast of China. Patients ≥18 years of age with a clinical diagnosis of ischemic stroke (15) confirmed by head computed tomography or magnetic resonance imaging were consecutively enrolled between March 2019 and January 2020. The following patients were excluded: patients with edema of the extremities, diagnosis of cancer, or presence of a cardiac pacemaker, and those with missing data.

This study was conducted in compliance with the principles of the Declaration of Helsinki. Study and the study protocol was approved by the ethical committee of the First Hospital of Jilin University (approval number: 2020-669). Written informed consent was obtained from all of the participants.

### 2.2 Definitions

MetS was determined based on algorithms proposed by the International Diabetes Federation (16). A diagnosis of MetS was based on the presence of at least three of the following five cardiovascular risk factors: (1) abdominal obesity: defined as a waist circumference (WC) of at least 90 cm for men and 80 cm for women; (2) triglyceride (TG) levels of at least 1.7 mmoL/L or undergoing treatment for hypertriglyceridemia; (3) high-density lipoprotein cholesterol (HDL-C) below 1.0 mmoL/L in men or 1.3 mmoL/L in women or undergoing treatment for reduced HDL-C; (4) hypertension, defined as a systolic blood pressure of at least 130 mmHg and/or a diastolic blood pressure of at least 85 mmHg or undergoing antihypertensive drug treatment; (5) fasting plasma glucose of at least 6.1 mmol/L (110 mg/dL) or undergoing treatment with a hypoglycemia agent.

### 2.3 Data collection and measurements

Data on patient socio-demographic characteristics, history of smoking and alcohol use, comorbidities, and stroke severity assessed by National Institute of Health stroke scale were retrieved from the electronic medical records. Smoking status was defined as smoking at least 5 cigarettes per day for more than 1 year. Drinking status was defined as drinking at least 50 g of alcohol per day for more than 1 year. BP of the non-hemiplegic side was measured after the patient rested for 5 min in the sitting position, with a digital sphygmomanometer (Omron HBP-9020). The mean value of two consecutive measurements with 1 minute intervals was taken as the final result.

Anthropometric measurements including body weight and height were conducted using an electronic scale with the patient wearing light clothing and without shoes according to standard protocols, with a single assessment recorded nearest 0.5 kg and 1 cm, respectively. BMI was calculated as weight (kg)/height (m^2^). According to the Working Group on Obesity in China, cut-off points for overweight and obesity were 24.0 kg/m^2^ and 28.0 kg/m^2^, respectively, for both sexes (17). WC was measured at the umbilical level using a non-stretchable tape to the nearest 0.1 cm. Biochemical indexes were assessed with venous blood samples collected after at least 12 h fasting overnight. VFA was measured using bioelectrical impedance analysis (BIA, Inbody S10, Biospace, Seoul, Korea) with the subject in the supine position at the same day. Visceral obesity was defined as a VFA ≥ 100 cm^2^ (18). All anthropometric parameters, body composition and laboratory data were obtained from participants within 48 h of hospitalization and measured by a trained stroke health manager (19).

### 2.4 Statistical analysis

Data were expressed as mean with standard deviation (SD) or median with interquartile range for normal and skewed distributions, respectively. Differences were analyzed using the Student’s *t*-test and the Mann–Whitney U-test, as appropriate. Categorical variables were presented as frequencies with proportions and compared using the chi-square test. A binary logistic regression model was used to estimate the odds ratio (OR) and 95% confidence interval (CI) of MetS risk factors while adjusting for related covariates. Receiver operating characteristic (ROC) curve and the area under the ROC curve (AUC) were used to determine the sex-specific cut-off values of VFA that indicated the presence of MetS. Cut-off values were obtained with the maximum Youden index (sensitivity + specificity − 1). A two-tailed *p* value <0.05 was considered statistically significant. All statistical analyses were performed using IBM Statistical Package for Social Science (SPSS) version 22.0 (SPSS, Inc., New York, United States).

## 3 Results

A total of 851 patients with ischemic stroke were included in the study. Mean age was 60.4 ± 11.2 years and 76.3% of patients were man (Table 1). The overall prevalence of MetS was 43.4%, and prevalence among women was significantly higher than that among men (57.9% vs. 38.8%, *p* < 0.001). Patients diagnosed with MetS were less frequently smoking and drinking, had significantly higher mean VAF (114.5 ± 29.8 cm^2^ vs. 79.9 ± 27.4 cm^2^, *p* < 0.001), BMI (26.8 ± 3.4 kg/m^2^ vs. 24.1 ± 3.1 kg/m^2^, *p* < 0.001), and WC (85.2 ± 9.3 cm vs. 79.6 ± 7.9 cm, *p* < 0.001) compared to those without MetS. The prevalence of hypertension, diabetes, dyslipidemia, and coronary heart disease, and levels of BP, triglycerides, FPG was significantly higher among those with MetS than among those without MetS.

**Table 1:** Demographic and clinical characteristic of ischemic stroke patients by MetS status.

	Total *N* = 851	With MetS *N* = 369	Without MetS *N* = 482	*p*-value
Age (years)	60.4 ± 11.2	60.1 ± 10.7	60.6 ± 11.6	0.553
Female, *n* (%)	202 (23.7)	117 (31.7)	85 (17.6)	<0.001
Smoking, *n* (%)	444 (52.2)	166 (45.0)	278 (57.7)	<0.001
Alcohol use, *n* (%)	407 (47.8)	155 (42.0)	252 (52.3)	0.003
NIHSS, median (IQR)	2 (1, 4)	2 (0, 5)	1.85 (1.24, 2.53)	0.181
Anthropometric indices				
VFA (cm^2^)	94.9 ± 33.2	114.5 ± 29.8	79.9 ± 27.4	<0.001
BMI (kg/m^2^)	25.3 ± 3.5	26.8 ± 3.4	24.1 ± 3.1	<0.001
WC (cm)	82.0 ± 9.0	85.2 ± 9.3	79.6 ± 7.9	<0.001
Comorbidities, *n* (%)				
Stroke	253 (29.7)	117 (31.7)	136 (28.2)	0.269
Hypertension	539 (63.3)	323 (87.5)	216 (44.8)	<0.001
Diabetes	266 (31.3)	205 (55.6)	61 (12.7)	<0.001
Dyslipidemia	356 (41.8)	181 (49.1)	175 (36.3)	<0.001
Coronary heart disease	97 (11.4)	51 (13.8)	46 (9.5)	0.052
Atrial fibrillation	34 (4.0)	14 (3.8)	20 (4.1)	0.793
SBP (mmHg)	145.1 ± 18.3	148.9 ± 16.5	142.1 ± 19.1	<0.001
DBP (mmHg)	85.3 ± 11.0	87.5 ± 11.1	83.6 ± 10.6	<0.001
Triglycerides (mmol/L)	1.76 ± 1.16	2.11 ± 1.23	1.48 ± 1.02	<0.001
HDL-C (mmol/L)	1.05 ± 0.25	0.99 ± 0.22	1.09 ± 0.26	<0.001
FPG (mmol/L)	6.12 ± 2.19	7.08 ± 2.50	5.38 ± 1.58	<0.001

NIHSS, National Institute of Health stroke scale; IQR, interquartile range; VFA, visceral fat area; BMI, body mass index; WC, waist circumference; SBP, systolic blood pressure; DBP, diastolic blood pressure; HDL-C, high-density lipoprotein cholesterol; FPG, fasting plasma glucose.

As shown in Table 2, after adjusting for potential confounders, women were more likely to have MetS than men (OR = 2.864, 95%CI: 1.837 to 4.464; *p* < 0.001). Visceral obesity according to VFA (OR = 7.449, 95%CI: 5.307 to 10.457; *p* < 0.001), being overweight (OR = 2.746, 95%CI: 1.863 to 4.047; *p* < 0.001) or obesity (OR = 6.002, 95%CI: 3.648 to 9.876; *p* < 0.001) were significantly associated with a high risk of MetS.

**Table 2:** Multivariate logistic regression model for the association between risk factors and MetS in ischemic stroke patients.

Variable	OR (95%CI)	95%CI	*p*-value
Age (≥60 years)	0.956	0.679–1.347	0.798
Sex (female)	2.864	1.837–4.464	<0.001
Smoking	0.787	0.536–1.155	0.220
Alcohol use	1.129	0.748–1.704	0.564
History of stroke	1.194	0.834–1.707	0.333
Coronary heart disease	0.903	0.541–1.508	0.696
Visceral obesity	7.449	5.307–10.457	<0.001
BMI (kg/m^2^)			
Overweight (24.0–27.9)	2.746	1.863–4.047	<0.001
Obesity (≥28)	6.002	3.648–9.876	<0.001

OR, odds ratio; CI, confidence interval; VFA, visceral fat area; BMI, body mass index.

**Figure 1:**
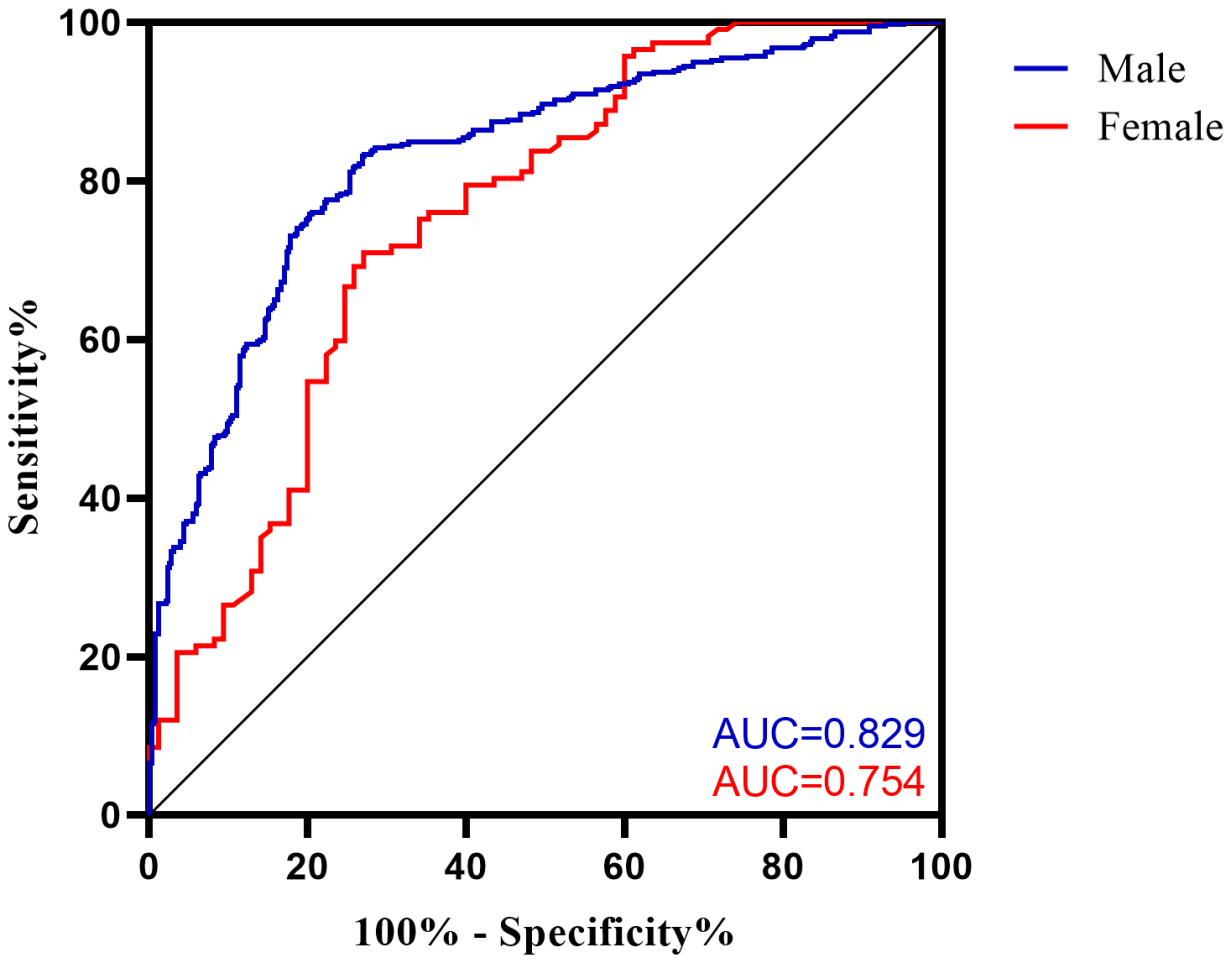
Receiver operating characteristic (ROC) curves for VFA cut-off values to discriminate MetS in ischemic stroke patients. AUC, area under the curve.

Sex-specific VFA cut-off values are presented in Table 3, with an area under the curve of 0.829 (95%CI: 0.797 to 0.861, *p* < 0.001) in men and 0.754 (95%CI: 0.684 to 0.823, *p* < 0.001) in women. The optimal VFA cut-off of 104.3 cm^2^ for men and 94.1 cm^2^ for women had a 73.0% sensitivity and 83.1% specificity and a 70.9% sensitivity and 72.9% specificity, respectively, to predict MetS (Figure 1).

**Table 3:** Sex specific cut-off values of VFA in predicting MetS in patients with ischemic stroke.

	VFA cut-off (cm^2^)	Sensitivity (%)	Specificity (%)	AUC	95%CI	*p*-value
Female	94.1	70.9	72.9	0.754	0.684–0.823	<0.001
Male	104.3	73.0	83.1	0.829	0.797–0.861	<0.001

VFA, visceral fat area; AUC, area under the curve; CI, confidence interval.

## 4 Discussion

This study revealed that MetS affected nearly a half of patients with ischemic stroke, with a higher prevalence among women and overweight or obese individuals. To the best of our knowledge, this is the first study to investigate the predictive value of VFA for MetS in patients with ischemic stroke. VFA of 104.3 cm^2^ and 94.1 cm^2^ were thresholds for predicting MetS in men and women, respectively.

The prevalence of MetS defined by the IDF criteria in our study was 43.4%, which is consistent with the results of other cross-sectional studies of patients with ischemic stroke in East China (6, 20). The reason for this high prevalence may be that each component of MetS is associated with an increased risk of stroke. However, the prevalence is still lower than that in other studies conducted in Europe (21, 22), although this may be explained by the fact that these studies included more women. Our study also confirmed that women are 2.86 times more likely to develop MetS than men. Changes in postmenopausal hormonal levels have been associated with the occurrence of MetS. Loss of the protective effects of estrogen and increased circulating androgens lead to increased insulin resistance, and the development of obesity, abnormal lipid metabolism, and other metabolic diseases (23, 24).

Interestingly, the proportion of smoking and alcohol use was higher among patients without MetS. The reason is that the proportion of males in this group of patients is higher, and according to WHO, the proportion of tobacco use and alcohol consumption among adult man is much higher than that of females. After adjusting for gender, the difference between these two groups was not statistically significant. Overweight and obese patients with ischemic stroke defined by BMI, were more likely to have MetS in our study. The main manifestation of obesity is excessive accumulation and abnormal distribution of fat in the body. Adipose tissue has a complex and highly active metabolic endocrine function, and can secrete a variety of adipokines, including adiponectin which plays an important role in protecting against insulin resistance/diabetes and atherosclerosis (25). Serum adiponectin concentration in obese patients was significantly lower than that in normal people (26). As an important mediator between obesity and cardiovascular disease, it adiponectin is associated with MetS through cardiometabolic risk factors. Increased visceral fat has been found to be associated with decreased total adiponectin levels (27). Therefore, VFA may be a useful anthropometric index for predicting the risk of MetS (28, 29). This hypothesis is also supported by the results of our study.

Previous studies have shown that mean VFA in men is higher than that in women (30). The lower VFA threshold in women supports this finding. Therefore, differences between the sexes should be considered when using VAF to predict obesity or related complications. Most previous studies have explored the cut-off value of VFA in predicting MetS among patients with type 2 diabetes, so it is difficult to compare the results of this study with previous research. Lee et al. found that the optimal VFA cut-off value increased with age, particularly in women (31). It is possible that the lack of stratification by age contributed to the slightly lower but acceptable sensitivity of the cut-off value for women in this study. At the same time, the different diagnostic criteria for MetS will also have an impact on the results of the study.

The main limitation of this study is the use of a cross-sectional study design which cannot establish a causal relationship between VFA and MetS among patients with ischemic stroke. Secondly, because there were fewer female patients, it was not possible to stratify according to age. Finally, the study was conducted at a single center so the generalization of the results to patients in other geographical regions should be approached with caution. Further multi-centered studies are required.

## 5 Conclusion

This study showed that approximately 43.4% of patients with ischemic stroke had MetS. Female, patients with large areas of visceral fat and high BMI were more likely to have MetS. This study is the first to investigate sex-specific optimal VFA cut-off values in patients with ischemic stroke with MetS. The predicted optimal VFA thresholds were 104.3 cm^2^ for men and 94.1 cm^2^ for women. The VFA by BIA may be a useful target for interventions to improve MetS, and incorporating the VFA measurements into regular physical examination may contribute to the early identification of MetS and the prevention of cerebrovascular disease.

## Data availability statement

The raw data supporting the conclusions of this article will be made available by the authors, without undue reservation.

## Ethics statement

The studies involving humans were approved by the ethical committee of the First Hospital of Jilin University (approval number: 2020-669). The studies were conducted in accordance with the local legislation and institutional requirements. The participants provided their written informed consent to participate in this study.

## Author contributions

XL: Formal analysis, Writing – original draft. JW: Formal analysis, Writing – review & editing. HS: Data curation, Writing – review & editing. DL: Data curation, Writing – review & editing. XY: Writing – review & editing. ZL: Conceptualization, Methodology, Supervision, Writing – review & editing.
